# Impact of Tick-Borne *Orthoflaviviruses* Infection on Compact Human Brain Endothelial Barrier

**DOI:** 10.3390/ijms26052342

**Published:** 2025-03-06

**Authors:** Felix Schweitzer, Tamás Letoha, Albert Osterhaus, Chittappen Kandiyil Prajeeth

**Affiliations:** 1 Research Center for Emerging Infections and Zoonoses, University of Veterinary Medicine, 30559 Hannover, Germany; felix.schweitzer@tiho-hannover.de (F.S.); albert.osterhaus@tiho-hannover.de (A.O.); 2 Center for Systems Neuroscience (ZSN), 30559 Hannover, Germany; 3 Pharmacoidea Ltd., H-6726 Szeged, Hungary; tamas.letoha@pharmacoidea.eu

**Keywords:** tick-borne encephalitis virus, Langat virus, blood–brain barrier, neuroinvasion, cerebral microvascular endothelial cells, laminin-binding protein

## Abstract

Tick-borne encephalitis remains a significant burden on human health in the endemic areas in Central Europe and Eastern Asia. The causative agent, tick-borne encephalitis virus (TBEV), is a neurotropic virus belonging to the genus of *Orthoflavivirus*. After TBEV enters the central nervous system (CNS), it mainly targets neurons, causing encephalitis and leading to life-long disabilities, coma and, in rare cases, death. The neuroinvasive mechanisms of orthoflaviviruses are poorly understood. Here we investigate the mechanism of TBEV neuroinvasion, hypothesizing that TBEV influences blood–brain barrier (BBB) properties and uses transcellular routes to cross the endothelial barrier and enter the CNS. To test this hypothesis, we employed an in vitro transwell system consisting of endothelial cell monolayers cultured on insert membranes and studied the barrier properties following inoculation to tick-borne orthoflaviviruses. It was shown that TBEV and closely related but naturally attenuated Langat virus (LGTV) crossed the intact endothelial cell monolayer without altering its barrier properties. Interestingly, transendothelial migration of TBEV was significantly affected when two cellular surface antigens, the laminin-binding protein and vimentin, were blocked with specific antibodies. Taken together, these results indicate that orthoflaviviruses use non-destructive transcellular routes through endothelial cells to establish infection within the CNS.

## 1. Introduction

Tick-borne encephalitis (TBE), caused by a virus (TBEV) belonging to the genus *Orthoflavivirus,* is a major health concern in endemic areas of Central Europe and Far-East Asia. In recent years, the incidence of the disease increased not only in the endemic areas, but also in regions where the disease was rare or not reported before. This could be attributed to changing climatic conditions, allowing the spread of vectors capable of transmitting the disease [1,2,3]. Serological studies indicate that 70–95% of human infections in endemic regions would go unnoticed or be asymptomatic [1]. Nevertheless, TBEV is highly neurotropic and causes a biphasic illness in affected individuals starting with a febrile illness, which either resolves in few days or can manifest itself with encephalitis of varying severity, leading to lifelong disabilities, coma or even death. Further, post-encephalitic syndrome can develop with long-term neurological symptoms such as cognitive impairments or behavioral changes, in addition to other long-lasting health problems [4,5]. Diagnosis of the disease by serology can be challenging since virus-specific antibody detection can lead to inconclusive results due to antibody cross-reactivity with other orthoflaviviruses. A reverse transcriptase polymerase chain reaction (RT-PCR)-based diagnosis is possible at the beginning of the disease. As there is no specific antiviral therapy, apart from prevention of contact to ticks, specific intervention can only be accomplished by preventive vaccination, while application of symptom-relieving medication remains the only treatment option [6,7].

To establish symptomatic infection, TBEV must reach from peripheral sites of infection into the central nervous system (CNS), which is protected by the compact blood–brain barrier (BBB). It is widely accepted that TBEV uses a blood-to-brain route to enter the CNS. Most of the knowledge related to neuropathogenesis and immunity to TBEV infections has come from animal infection models. Interestingly, despite protective immunity from symptomatic disease induced by immunization, we have observed TBEV RNA copies and signs of inflammation in the CNS in a mouse model of lethal TBEV challenge [8]. However, the mechanism by which TBEV can cross the blood–brain barrier is not well understood. The BBB is mainly formed of vascular endothelial cells, which constitute capillary networks that can have different structural forms. The BBB differs from peripheral vascular endothelia as it is formed by continuous, non-fenestrated endothelia [9,10]. On the basal side of the endothelial cell layer is a basal lamina that is mainly formed by collagen, laminin and heparan sulfate [11]. To form an effective barrier, the endothelial cells sitting on this basal lamina are held together by tight junctions. These are associated with several intra- and extracellular proteins, like claudin-5, zonula occludens protein (ZO-)1 and occludin. Tight junctions, in general, are important for barrier integrity, and leaky tight junctions may lead to a breakdown of the BBB and, with that, a loss of function. Certain viruses are known to alter the expression of tight junction proteins and thus influence the barrier properties of endothelial cells [12,13].

Apart from barrier breakdown, viruses have evolved mechanisms to exploit transcellular routes to cross the BBB without largely affecting the barrier properties. Transcytosis is one such mechanism by which the viruses enter endothelial cells on the luminal side and then virus-containing vesicles are translocated across the cell and released into the brain parenchyma. This non-disruptive mechanism is also used by other neurotropic pathogens to establish initial infection within the CNS [14]. In addition to this, viruses can cross the BBB by entering peripheral immune cells, which then infiltrate into the CNS by crossing the BBB (“Trojan Horse Mechanism”). Alternatively to haematogenic routes, viruses could bypass the BBB by infecting peripheral nerves and using a retrograde neuronal infection route to the CNS, or by entering the CNS via translocation through the blood–cerebrospinal fluid (CSF) barrier [13,15,16]. As for encephalitic orthoflaviviruses in general (including TBEV), the mechanisms underlying CNS entry are not completely clear, although it is assumed that TBEV enters the CNS more likely on a blood-to-brain transmission route, rather than through infection of peripheral nerves. Underlying this assumption is the idea that TBEV is able to cross the BBB, and that TBEV infection has been shown to lead to a damaged blood–CSF barrier [4,17]. While there was no BBB breakdown detected during the viraemic phase in experimentally infected mice, BBB integrity seems to be reduced after TBEV was detected in the brain. This suggests that TBEV can enter the CNS through an intact BBB [18,19,20].

To further study the events during TBEV infection at the BBB, we established an in vitro model using an immortalized human cerebral microvascular endothelial cell line (hCMEC/D3 [21]) cultured on the apical side of transwell inserts. This model has been widely used for understanding BBB function and for investigating mechanisms of drug delivery across the BBB [9,20,22,23,24,25,26,27]. Using this model, we demonstrate the ability of two tick-borne orthoflaviviruses, TBEV and its close relative, Langat virus (LGTV), to cross the endothelial barrier. We further studied the effect of viral inoculation on the barrier properties by determining virus release on both sides of the endothelial barrier via viral RNA detection. To gather further insights into mechanisms involved in translocation of tick-borne orthoflaviviruses, we implemented inhibitors of intracellular transport and antibodies directed against potential receptors in our study.

## 2. Results

### 2.1. Barrier Integrity Is Not Affected by Virus Inoculation

**Figure 1:**
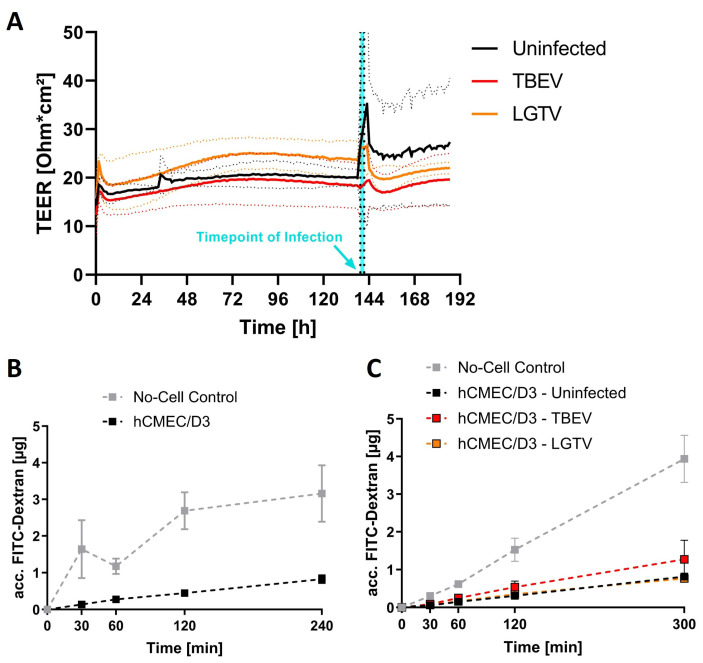
Effects of TBEV infection on barrier properties of hCMEC/D3 cells (**A**) TEER values of mock- and tick-borne encephalitis virus (TBEV)- (red) and Langat virus (LGTV)-inoculated (orange) hCMEC/D3 cells cultured on transwell inserts were measured in real-time before and after inoculation using cellZscope^®^. Data presented are the mean of *n* = three replicates (continuous lines) ± SEM (dotted lines). (**B**) Accumulation of apically added 70 kDa FITC-dextran in the basolateral compartment of hCMEC/D3 transwell system was measured in barrier integrity assays (*n* = 6). (**C**) Similarly, fluorescence associated with FITC-dextran leakage in the basolateral compartments at 48 h after inoculation with TBEV (*n* = 2) and LGTV (*n* = 2) was quantified and compared with uninfected hCMEC/D3. To assess barrier-free permeability control, inserts without endothelial cells were used (*n* = 2). Data presented as mean ± SEM.

To study viral translocation across the endothelial barrier, hCMEC/D3 was cultured on transwell inserts to form compact monolayers. Compactness of the endothelial monolayers was assessed in real-time by measuring transendothelial electrical resistance (TEER) using a cellZscope+^®^ (nanoAnalytics, Münster, Germany). A plateau in TEER of hCMEC/D3 cell monolayers was reached 5–6 days after seeding, with TEER values ranging between 15–25 Ωcm^2^ (Figure 1A). At this point, integrity of the endothelial barrier was assessed by checking the leakage of high molecular weight FITC-dextran (70 kDa) through the compact endothelial monolayer. Confirming the barrier integrity, we saw only a negligible amount (less than 0.5 µg) of FITC-dextran leakage even after 60 min (Figure 1B). Subsequently, each hCMEC/D3 monolayer was inoculated with 2.5 × 10^5^ TCID_50_ of TBEV or LGTV and, over the period of two days post inoculation (dpi), TEER values were measured and compared with that of mock-infected inserts. No significant difference in the TEER was observed between mock and virus-infected endothelial monolayers (Figure 1A).

Furthermore, FITC-dextran barrier integrity assays using virus-inoculated inserts further confirmed that no loss of integrity of endothelial barrier at 48 h post inoculation (hpi) took place, as accumulation of FITC-dextran in the basolateral compartment remained below 0.5 µg within 60 min of FITC-dextran incubation, which corresponds to the levels of leakage before inoculation (Figure 1C).

### 2.2. Virus Translocation Across Compact hCMEC/D3 Barrier

**Figure 2:**
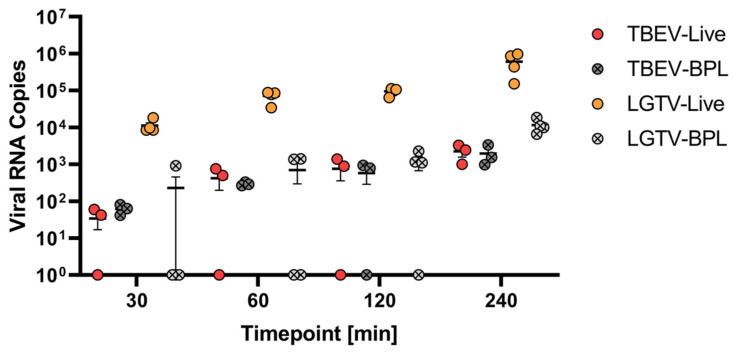
Detection of viral RNA via RT-qPCR of RNA collected from basolateral supernatants at different time points (30, 60, 120 and 240 min) after inoculation with live tick-borne encephalitis virus (TBEV-Live) and Langat virus (LGTV-Live), as well as BPL-inactivated TBEV (TBEV-BPL) and inactivated LGTV (LGTV-BPL). Individual values of *n* = three (TBEV-Live and TBEV-BPL) and *n* = four (LGTV-Live and LGTV-BPL) replicates, respectively, are presented with the mean ± SEM.

In the subsequent experiments, we tested the ability of both TBEV and LGTV to cross the endothelial barrier. In addition to live viruses, we also tested the ability of β-propiolactone (BPL)-inactivated viruses to cross the endothelial barrier. For this, either live or BPL-inactivated TBEV or LGTV were added to the apical side of the transwell inserts, and supernatants collected from the basolateral compartments at 30, 60, 120 and 240 min after inoculation were screened for the presence of viral RNA copies. Interestingly, live LGTV more readily crossed the endothelial barrier, with approximately 10^4^ viral RNA copies detected within 30 min post inoculation. However, TBEV was less efficient in crossing the compact endothelial barrier as less than 10^2^ RNA copies were detected after 30 min, which gradually increased to approximately 10^3^ copies by 240 min. In both cases, inactivated virus particles were less efficient in crossing the endothelial barrier. These results indicate that both infectious TBEV and LGTV can cross the endothelial barrier, although with different efficacies (Figure 2).

**Figure 3:**
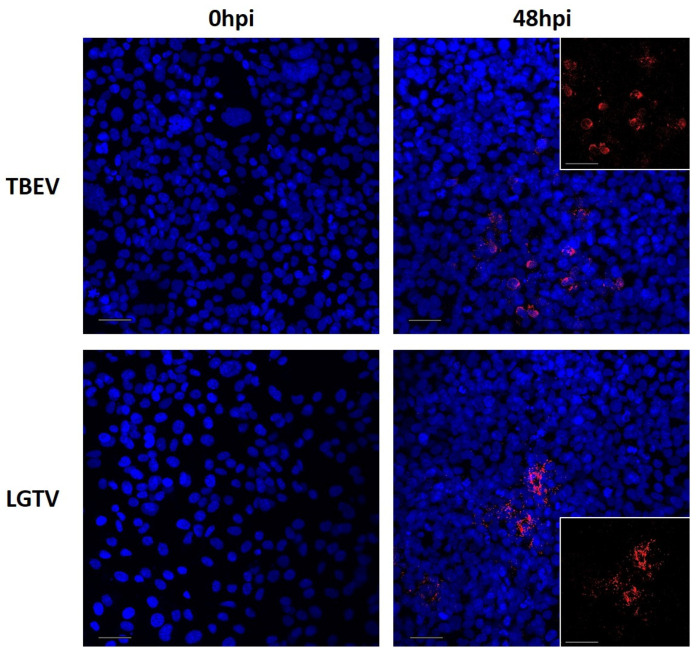
Immunocytochemistry to determine tick-borne encephalitis virus (TBEV) and Langat virus (LGTV) infection of hCMEC/D3. Cells were cultured on glass coverslips, inoculated with the respective virus and fixed at different timepoints after inoculation. Antibodies targeting TBEV E-protein (red) and DAPI-based probes (nuclei, blue) were used for immunofluorescence staining. Representative images for each timepoint of fixation (0 hpi, 48 hpi), small images show red channel only. Scalebars represent 50 µm.

To assess the susceptibility of hCMEC/D3 to TBEV and LGTV infection, we fixed confluent monolayer at various timepoints post virus inoculation and performed immunostaining for the detection of TBEV or LGTV E-protein antigen. However, at 48 hpi, E-proteins were detected in a few cells that had been exposed to TBEV and LGTV. Hence, it is likely that TBEV and LGTV infects and replicates within hCMEC/D3 cells with relatively low efficiency (Figure 3).

### 2.3. Influence of Modulators of Endocytosis on LGTV Translocation

**Figure 4:**
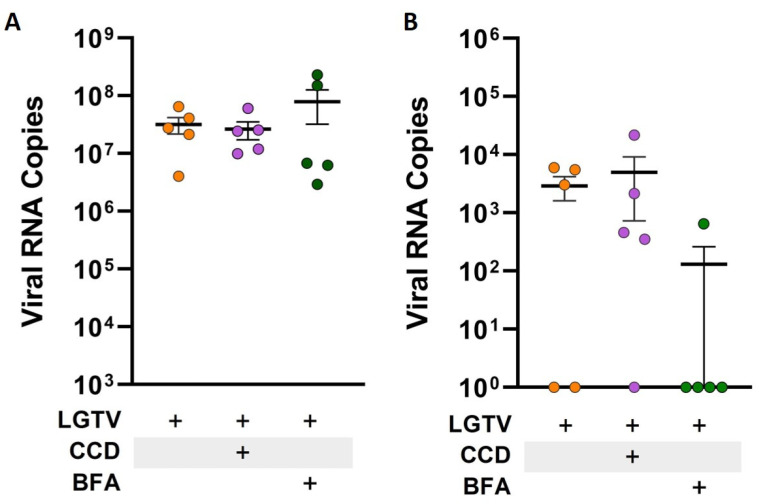
Detection of viral RNA via RT-qPCR of RNA collected from apical (**A**) and basolateral (**B**) supernatants one hour after inoculation with Langat virus (LGTV). Cells were either pre-treated with cytochalasin D (CCD), brefeldin A (BFA) or left untreated. Data points are from five replicates (*n* = 5) and presented as mean ± SEM.

To determine whether the viruses reach the basolateral compartments via transcellular routes or by leakage through intercellular spaces, we performed transwell experiments in the presence and absence of pharmacological agents known to impact the vesicular transport, such as brefeldin A (BFA) and cytochalasin D (CCD). For this, transwell inserts with compact endothelial monolayers were treated with BFA or CCD two hours prior to the addition of LGTV onto the apical side of the insert. Following inoculation of LGTV in the presence or absence of the inhibitors, supernatants were collected from both apical and basolateral compartments, and viral RNA copies were quantified. Pre-treatment of the endothelial monolayer with CCD did not have a major impact on LGTV crossing the endothelial barrier and reaching basolateral compartments. Interestingly, BFA treatment of endothelial cells prevented LGTV from crossing through the endothelial barrier in four out of five replicates. Nevertheless, this did not yield statistical significance for the reason that, in two out of five replicates with untreated endothelial monolayer, no LGTV RNA was detected in basolateral compartments (Figure 4). Hence, it is more likely that LGTV use transcellular routes to cross the endothelial barrier.

### 2.4. Blocking of Laminin-Binding Protein and Vimentin Reduces Translocation of TBEV Across Endothelial Barrier

**Figure 5:**
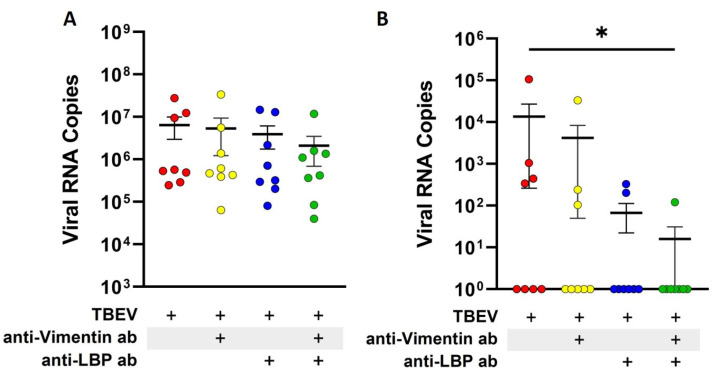
Detection of viral RNA via RT-qPCR of RNA collected from apical (**A**) and basolateral (**B**) supernatants after one-hour inoculation with tick-borne encephalitis virus (TBEV). Cells were either treated with antibodies directed against laminin-binding protein (anti-LBP ab) or vimentin (anti-Vimentin ab) for two hours before inoculation. Individual values of eight replicates (*n* = 8) are presented with the mean ± SEM. * represents a *p*-value < 0.05.

Laminin-binding protein (LBP) is a known receptor for TBEV E-protein [28]. Additionally, vimentin, a component of the cytoskeleton, has also been shown to contribute to the viral entry of other orthoflaviviruses such as Japanese encephalitis virus (JEV) [29]. To assess the involvement of surface receptors in transcellular migration of TBEV across the endothelial barrier, we performed antibody-mediated receptor blocking studies. First, we confirmed the expression of LBP and vimentin on the surface of hCMEC/D3 cells (Supplementary Figure S1). Subsequently, LBP and vimentin on the surface of endothelial cells were blocked with the respective specific antibodies before TBEV was added to the endothelial monolayer cultured on transwell inserts. Viral RNA copies in the culture supernatants from apical and basolateral compartments were quantified to assess the effect of receptor blocking. After one hour of inoculation, no significant changes were detected in the TBEV RNA copies in the apical supernatants, irrespective of whether LBP or vimentin were blocked (Figure 5A). Similar to previous experiments, translocation of TBEV through the hCMEC/D3 monolayer into the basolateral compartment was less efficient as viral RNA was below detection in four out of eight replicates. No striking effect was seen when vimentin alone was blocked on the endothelial cells, whereas a slight decrease was observed when LBP was blocked. This, however, did not yield statistical significance. Interestingly, a significant decrease in TBEV translocation through hCMEC/D3 cells was observed when a combination of LBP and vimentin blocking antibodies were used. In this case, no viral RNA was detected in the basolateral compartments in seven out of eight replicates. These results suggest a combined role of vimentin and LBP in the binding, uptake and translocation of TBEV across the endothelial cell barrier (Figure 5B).

## 3. Discussion

In this study, we demonstrate that both TBEV and LGTV can cross an in vitro compact human brain microvascular endothelial cell barrier without compromising the barrier properties, although with lower efficiency within the initial hours of virus inoculation. This process occurs without virus replication within endothelial cells and thus most likely involves translocation of viruses through endocytic vesicles from the apical side into the basolateral compartments of the model. This mechanism, generally referred to as transcytosis, apparently is an efficient mechanism to bypass the BBB and is used by several pathogens to establish infection in the CNS without disrupting the compact barrier [14,30,31,32].

Although TBEV is a neurotropic virus, the mechanisms by which it gains access to the CNS are rather unclear. Interestingly, in our testing, naturally attenuated LGTV more readily crossed the compact endothelial barrier than TBEV. The reason for this is not clear. LGTV belonging to the TBEV serocomplex has a considerable amount (83–95%) of viral genome sequence similarity with TBEV [33,34]. It is likely that LGTV may interact with different cellular components that assist its translocation across the endothelial barrier compared to TBEV. Furthermore, at this moment, it is unclear if this is an in vitro artifact or also occurs in vivo, as there have been only limited studies comparing the neuroinvasion of TBEV and LGTV in vivo in experimental animal infection models. It is noteworthy that several factors, including the host response to the virus, are critical in determining the infectivity and pathogenicity of TBEV in vivo. A study on LGTV infection in mice indicates that local and systemic type-I interferon responses prevent LGTV from entering into the CNS [19]. One major limitation of an in vitro transwell model is that the integrity and barrier properties achieved by endothelial cell culture do not replicate the higher transendothelial resistance observed in physiological BBB in vivo [23]. Hence, it is challenging to distinguish between the leakage of viruses through intercellular spaces and crossing of the viruses using transcellular routes. However, in our model, we could confirm that the barrier attained by endothelial culture on transwell inserts is largely impermeable to high molecular weight (70 kDa) dextran molecules. Therefore, it is unlikely that viruses, which are considerably larger than these dextran molecules, cross the endothelial barrier through paracellular leakage unless the expression of tight junction proteins is altered by infection. Consistent with our findings, a previous study has reported that TBEV is capable of crossing a human brain endothelial cell barrier without altering the expression of tight-junction proteins [20]. Interestingly, in our study, the barrier integrity remained intact even at 48 h post inoculation, at which virus antigen within the endothelial cells was first observed. Unlike hCMEC/D3 cells, both TBEV and LGTV infect and efficiently replicate in A549 cells (human lung epithelial cells) and induce cytopathic effects. This indicates that endothelial cells are probably not equipped with the cellular machinery that supports TBEV replication and release, which otherwise could have deleterious consequences.

Pharmacological agents CCD and BFA, which modulate endocytosis and vesicular transport, respectively, were used to evaluate the role of endocytic pathways in this process. CCD disrupts actin polymerization and thus affects the reorganization of the cytoskeleton. Previously, it had been shown that cytochalasins also influence the uptake and release of some notable viral pathogens such as measles virus, SARS-CoV-2 and influenza virus [35,36,37], as well as other orthoflaviviruses such as Dengue virus (DENV) and West Nile virus (WNV) [38,39]. Here, no effects of cytochalasin D on apical to basolateral translocation of LGTV were observed. Alternatively, BFA triggers the collapse of the Golgi apparatus into the endoplasmic reticulum and thereby impacts the vesicular transport. Previous studies have shown that pre-treatment of cells with BFA or its addition during the first four hours of virus inoculation interferes with DENV and Zika virus (ZIKV) infection by blocking virus release from infected cells [32,38,40]. However, only a subtle effect of BFA in this process was observed in our study. Due to the low efficiency of LGTV transcytosis across the endothelial barrier, resulting in pronounced variations between the replicates, this did not yield statistical significance. Currently, it is unclear if the viral RNA detected on the basolateral compartment corresponds to infectious virus particles or only viral RNA transported across the endothelial cells. It has been shown that exosomes containing replicative LGTV RNA can introduce the virus to cells of the CNS, surpassing the necessity of viral replication in endothelial cells [41]. The addition of culture supernatants from the basolateral compartments resulted in infection of permissive cells, suggesting the presence of infectious virus particles (Supplementary Figure S2).

Profound knowledge related to the receptors used by neurotropic orthoflaviviruses to infect cells is currently lacking. Studies have shown that orthoflaviviruses such as DENV, WNV and ZIKV use αβ-integrins to mediate their infection [42,43,44,45]. For TBEV, laminin-binding protein, also known as 67 kDa laminin receptor, has been identified as a potential receptor that interacts with the viral E-protein [28,46]. Additionally, vimentin, an intermediate filament of the cytoskeleton also known to be expressed on the surface of endothelial cells [47], has also been shown to act as a receptor for DENV on endothelial cells [48,49]. It is likely that TBEV binds to certain surface receptors on endothelial cells, which subsequently mediate its uptake. Blocking experiments using specific antibodies directed against LBP and vimentin have provided further insights into the mechanism. When LBP or vimentin were blocked individually, no significant effect on TBEV transcytosis across the endothelial monolayer was observed. Interestingly, when both LBP and vimentin were blocked together, a marked decrease in the translocation of virus across the compact endothelial monolayer was seen. These findings suggest that there is redundancy in the usage of these proteins by TBEV. It is not clear if this mechanism is specific to endothelial cells. However, no effects of LBP or vimentin blocking on TBEV infectivity was observed for permissive cells such as A549 (Supplementary Figure S3).

In summary, TBEV is capable of breaching a compact endothelial barrier without significantly altering its barrier properties. This step might help in the initial entry of TBEV into the CNS and thus be crucial for the viral neuropathogenesis. Replication within the targeted cells combined with the induced neuroinflammatory response may eventually compromise the BBB and lead to a second wave of viruses infiltrating the CNS. Promising insights resulting from research assessing viral entry into the CNS could lead to better early treatments for related infectious diseases, and these may also have the potential to improve drug delivery into the brain for treatment of neurological diseases in general [50].

In conclusion, the data obtained in the in vitro BBB model add to the understanding of the mechanisms underlying the neuroinvasion of orthoflaviviruses.

## 4. Materials and Methods

### 4.1. Viruses

The source and propagation of TBEV (strain Neudoerfl) and LGTV (strain TP21) used in this study was previously described [8,51]. Briefly, viral stocks were generated by propagating them in A549 (TBEV) and VeroE6 (LGTV) cell lines, respectively, and titers were determined by tissue culture infectious dose (TCID_50_) assay on A549 (TBEV) and VeroE6 (LGTV) cells. Additionally, some of the viral stocks were inactivated by β-propiolactone (BPL, Merck Darmstadt, Germany) treatment. Briefly, viral stocks were incubated with BPL (0.1%) for 72 h at 4 °C, followed by BPL hydrolyzation by incubation at 37 °C for one hour. The viral suspension was filtered through amicon^®^ Ultra-15 30 k centrifugal filters (Merck Millipore, Darmstadt, Germany) and aliquots were generated.

### 4.2. Human Cerebral Microvascular Endothelial Cells

Commercially available human cerebral microvascular endothelial cell-line D3 (hCMEC/D3) was obtained from Merck KGaA (Darmstadt, Germany). Initially, cells were grown in type I rat tail collagen (RTC)-coated T75 culture flasks in EndoGRO^®^-MV complete culture medium (Merck Millipore, Darmstadt, Germany), supplemented with human fibroblast growth factor 2 (hFGF-2; 1 ng/mL; Merck Millipore, Darmstadt, Germany) (EGM+) until confluent. Cells were then harvested by trypsinization and transferred into fresh T75 culture flasks where they were grown until confluent, again. Subsequently, cells were passaged in a similar manner and, at the time of cell harvest, several aliquots of cells from each passage were transferred in EGM+ medium with 10% dimethyl sulfoxide (DMSO; Carl Roth, Karlsruhe, Germany) and stored at −150 °C until further use. For the experiments, endothelial cells were used up to passage 15.

### 4.3. In Vitro Transwell Barrier Model

To set up a compact human brain endothelial barrier model, hCMEC/D3 cells were cultured on RTC-coated polyester membrane of 24-well transwell inserts (pore size of 0.4 µm; Corning^®^ costar^®^, Corning, NY, USA), as essentially described by Weksler et al. [9]. For this, hCMEC/D3 cells were harvested from confluent cultures and the cells were seeded at a density of 5 × 10^4^ cells per insert on the apical side of the membrane. The following day, culture medium was changed, and inserts were transferred to a cellZscope-device (cellZscope+^®^, nanoAnalytics, Münster, Germany) and transendothelial electrical resistance (TEER) of inserts with hCMEC/D3 cells was determined by subtracting baseline values obtained from RTC-coated inserts without cells. Formation of a compact monolayer was indicated by a plateau in TEER values derived from tight cell layers with close cell–cell connections, and thus, barrier integrity can be assumed [52].

### 4.4. Barrier Permeability Assay

Barrier integrity of the endothelial cell monolayer was assessed by high molecule-weight particle permeability assay. For this, fluorescein isothiocyanate (FITC) coupled to 70 kDa dextran (FITC-dextran; Sigma Aldrich, St. Louis, MO, USA) was added to the apical side of inserts showing a desired TEER value plateau (>15 Ωcm^2^) at a final concentration of 1 mg/mL. Basolateral culture supernatants were subsequently collected at intervals of 0, 30, 60, 120, 240 and 300 min after FITC-dextran application, and concentrations of FITC-dextran were measured (excitation wavelength: 485 nm, emission wavelength: 535 nm; Infinite^®^ 200 Pro plate reader, TECAN Group, Männedorf, Switzerland). Absolute FITC-dextran concentration at each timepoint was quantified by measuring fluorescence of a 2-fold serial dilution of the applied FITC-dextran solution and generating a standard curve.

### 4.5. Virus Translocation Across hCMEC/D3 Monolayer Cultured on Transwell Inserts

To study virus translocation, hCMEC/D3 monolayer cultured on transwell inserts were used when the TEER values reached approximately 15 Ωcm^2^. Prior to the addition of inoculum, cells were incubated for two hours in serum-free EGM+ medium. To the apical side of the transwell membrane, 2.5 × 10^5^ TCID_50_ of TBEV or LGTV were added in 100 µL of serum-free EGM+, respectively. After one hour of virus inoculation at 37 °C and 5% CO_2_, 50 µL of the inoculum and supernatants from the basolateral compartments were collected into 450 µL of TRIzol^®^ reagent (life technologies^TM^, Carlsbad, CA, USA) and stored at −80 °C until further use. Inserts were thoroughly washed to remove the inoculum and were incubated for another hour at 37 °C and 5% CO_2_. At this point, 50 µL of supernatant were collected from apical and basolateral compartments into 450 µL of TRIzol^®^ reagent and stored at −80 °C until further use.

Essentially, similar to applications described previously [53], for conditions involving treatment with inhibitors, cells were incubated with either cytochalasin D (CCD; life technologies^TM^, Carlsbad, CA, USA) at a final concentration of 1 µg/mL, or brefeldin A (BFA; fisher scientific, Pittsburgh, PA, USA) at a final concentration of 3 µg/mL in serum-free EGM+, two hours prior to the addition of the inoculum. Similarly, for antibody-based blocking studies, cells were incubated with antibodies directed against either laminin-binding protein (LBP) or vimentin (for further information see Table 1) for two hours at a final concentration of 1 µg/mL in serum-free EGM+. In all these conditions, subsequent incubation with the virus inoculum was preformed in the presence of inhibitors or blocking antibodies by the addition of 2.5 × 10^5^ TCID_50_ of TBEV or LGTV in 10 µL of serum-free EGM+ to the respective solutions in the apical compartment.

### 4.6. RNA Extraction

For quantitative detection of virus, total RNA was extracted from supernatants in TRIzol^®^ reagent, following the manufacturer’s protocol (life technologies^TM^, Carlsbad, CA, USA). Briefly, RNA extraction was initiated by the addition of trichloromethane/chloroform (Carl Roth, Karlsruhe, Germany). After 15 min of incubation and subsequent centrifugation (15 min, 12,000× *g*, 4 °C) in a fixed rotor centrifuge (HEREAUS Fresco 17, Thermo Fisher Scientific, Waltham, MA, USA), the aqueous phase was transferred to fresh tubes and incubated with isopropanol (100%; Carl Roth, Karlsruhe, Germany) for 10 min at room temperature. Following centrifugation (10 min, 12,000× *g*, 4 °C), extracted RNA was washed by resuspension in ethanol (75%; Carl Roth, Karlsruhe, Germany), followed by centrifugation (5 min, 7500× *g*, 4 °C) and subsequent resuspension in nuclease-free water. Finally, extracted RNA solutions were heated at 60 °C for 10 min and solutions were stored at −20 °C until RT-PCR was performed the following day.

### 4.7. Virus Quantification RT-qPCR

Quantification of viral RNA was performed via one-step reverse transcriptase real-time PCR (RT-qPCR), using a OneStep RT-PCR kit (Qiagen^®^, Venlo, The Netherlands), as described previously [8,51]. TBEV and LGTV specific primers with the following sequences were used: TBEV forward primer (5′→3′ GGGCGGTTCTTGTTCTCC) and TBEV reverse primer (5′→3′ ACACATCACCTCCTTGTCAGACT), designed as described in [54]; and LGTV forward primer (5′→3′ AACGGAGCCATAGCCAGTGA) and LGTV reverse primer (5′→3′ AACCCGTCCCGCCACTC), designed as described in [55]. Real-time detection was facilitated by the usage of carboxy fluorescein (FAM) plus black hole quencher 1 (BHQ1) probe constructs, designed for TBEV (5′→3′ FAM-TGAGCCACCATCACCCAGACACA-BHQ1) as in [54] and for LGTV (5′→3′ FAM-AGAGACAGATCCCTGATGG-BHQ1) as in [55]. Viral RNA copy number was determined from a standard curve generated using TBEV and LGTV RNA standards [8]. RT-qPCR was performed using AriaMx Real-time PCR Systems (Agilent, Waldbronn, Germany).

### 4.8. Immunocytochemistry of hCMEC/D3 Inoculated with Virus

Immunocytochemistry (ICC) was performed in a comparative manner as described in Petry et al. [8]. Briefly, hCMEC/D3 were seeded on RTC-coated 12 mm glass coverslips at a density of 1 × 10^5^ cells per coverslip and incubated in EGM+ for up to 4 days at 37 °C. Subsequently, 5 × 10^5^ TCID_50_ of TBEV or LGTV were applied in 100 µL of serum-free EGM+ and cells were inoculated with viral solutions or serum-free EGM+ without virus for one hour at 37 °C. Afterwards, the inoculum was washed off and incubation was continued. At day one and two post infection, cells were fixed with paraformaldehyde (4% in PBS) for 20 min. After blocking with normal goat serum (3% in PBS-T; Thermo Scientific invitrogen, Carlsbad, CA, USA), cells were incubated with primary antibodies directed against tick-borne encephalitis virus E-protein, human laminin-binding protein or human vimentin (Table 1) for two hours at room temperature. Following this, primary antibody solutions were removed, and cells were washed thoroughly, before fluorescent-labelled secondary antibodies were applied, as described in Table 1. Finally, cell nuclei were stained by application of NucBlue^TM^ Live Cell Stain ReadyProbes^TM^ (Thermo Scientific invitrogen, Carlsbad, CA, USA) for 15 min at room temperature. Microscopy was performed using a Leica TCS SP5 confocal microscope and images were processed using Leica LAS software (Leica Application Suite X, Version 3.7.2.22383).

**Table 1:** Antibodies used in TBEV-virus inoculation assays and immunocytochemistry assays.

Primary Antibody					Secondary Antibody
Antigen/Target	Origin	Clonality	Dilution	Provider	
Tick-borne encephalitis virus E-protein	Mouse	Monoclonal (Clone 1786)	1:100	Matthias Niedrig [56]	Goat-anti-mouse IgG Alexa Fluor^®^ 568 (abcam, Cambridge, UK)
Human laminin-binding protein	Rabbit	Polyclonal	1:500	Merck, Darmstadt, Germany	Goat-anti-rabbit IgG Alexa Fluor^®^ 488 (Thermo Scientific invitrogen, Carlsbad, CA, USA)
Human Vimentin	Goat	Polyclonal (antiserum)	1:50	Merck, Darmstadt, Germany	Donkey-anti-goat IgG Alexa Fluor^®^ 488 (Thermo Scientific invitrogen, Carlsbad, CA, USA)

### 4.9. Statistical Analysis

For data display and analysis, GraphPad Prism Software 9 was used. Viral RNA copies of individual replicates are displayed together with the mean and standard error of the mean (SEM). Analysis of variance was performed as non-parametric *Kruskal–Wallis Test* with an Uncorrected Dunn’s Test for multiple comparisons, with *p*-values of <0.05 being considered significant (indicated with *).

## Data Availability

The data are presented as figures and supplementary figures, and any additional information supporting the conclusions of this manuscript will be made available by the authors on request.

## Supplementary Materials

The supporting information can be downloaded at https://www.mdpi.com/article/10.3390/ijms26052342/s1.

## Abbreviations

The following abbreviations are used in this manuscript:

BBB	Blood–brain barrier
BFA	Brefeldin A
BHQ1	Black hole quencher 1
BPL	β-propiolactone
CCD	Cytochalasin D
CNS	Central nervous system
CSF	Cerebrospinal fluid
DENV	Dengue virus
DMSO	Dimethyl sulfoxide
dpi	Days post inoculation
EGM+	EndoGRO^®^-MV complete culture medium supplemented with hFGF-2
E-protein	Envelope protein
FAM	Carboxy fluorescein
FITC	Fluorescein isothiocyanate
hCMEC/D3	Human Cerebral Microvascular Endothelial Cell Line (D3)
hFGF-2	Human fibroblast growth factor 2
hpi	Hours post inoculation
ICC	Immunocytochemistry
JEV	Japanese encephalitis virus
LBP	Laminin-binding protein
LGTV	Langat virus
PBS	Phosphate-buffered saline
PBS-T	Phosphate-buffered saline supplemented with 0.05% Tween20 reagent
RNA	Ribonucleic acid
RTC	Rat tail collagen
RT-PCR	Reverse transcription polymerase chain reaction
RT-qPCR	Reverse transcription real-time polymerase chain reaction
TBE	Tick-borne encephalitis
TBEV	Tick-borne encephalitis virus
TCID_50_	Tissue culture infectious dose
TEER	Transendothelial electrical resistance
WNV	West Nile virus
ZIKV	Zika virus
ZO-1	Zonula occludens protein 1
