# Supplementary material for: Impact of Tick-Borne Orthoflaviviruses Infection on Compact Human Brain Endothelial Barrier
Source: Int J Mol Sci. 2025 Mar 6;26(5):2342. doi: 10.3390/ijms26052342 (PMC11901142; doi:10.3390/ijms26052342)
Supplement: Supplementary file 1 [file ijms-26-02342-s001.zip › ijms-3492071-supplementary.pdf]

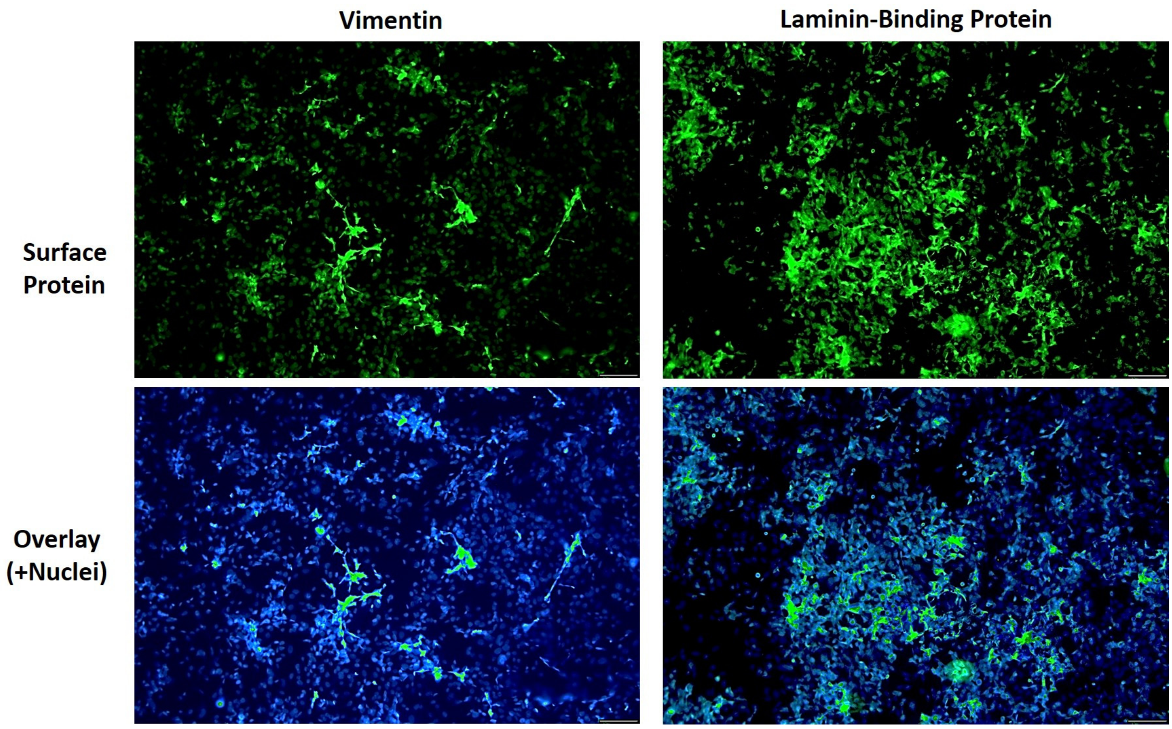

Figure S1: ICC of hCMEC/D3 for detection of LBP and vimentin surface expression. Scalebars represent 100μm.

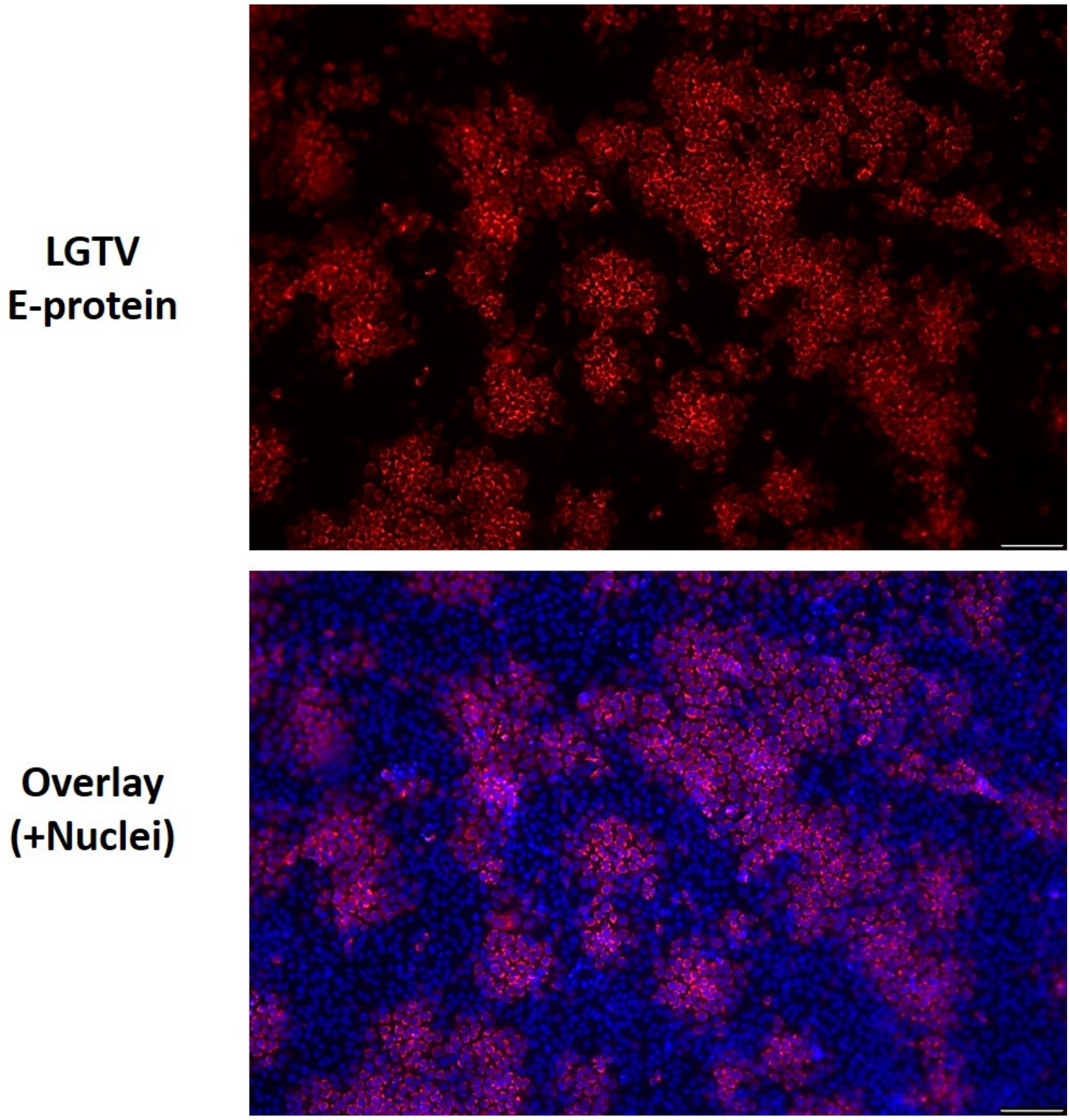

Figure S2: ICC of VeroE6 for virus detection in the basolateral compartment of hCMEC/D3 transwell insert cultures inoculated with LGTV. Scalebars represent 100µm.

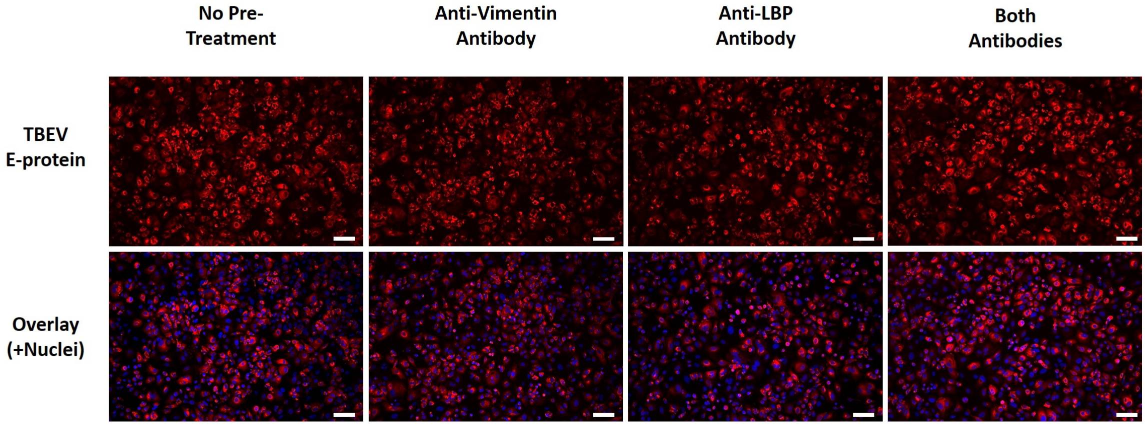

Figure S3: Immunofluorescence images of A549 cells at 48 h post inoculation with TBEV. Cells were either treated with antibodies directed against Vimentin, LBP or a combination of both for 2 h before virus inoculation. Subsequently, the cells were fixed and stained with specific antibodies to detect TBEV E-protein. Scalebars represent 100µm.
